# Blocking iASPP/Nrf2/M-CSF axis improves anti-cancer effect of chemotherapy-induced senescence by attenuating M2 polarization

**DOI:** 10.1038/s41419-022-04611-4

**Published:** 2022-02-21

**Authors:** Hao Liu, Dong Zhao, Huayi Li, Wenxin Zhang, Qingyu Lin, Xingwen Wang, Shanliang Zheng, Lei Zhang, Li Li, Shaoshan Hu, Ying Hu

**Affiliations:** 1 Department of Neurosurgery, Emergency Medicine Center, Zhejiang Provincial People’s Hospital, Affiliated to Hangzhou Medical College, 310000 Hangzhou, Zhejiang China; 2 School of Life Science and Technology, Harbin Institute of Technology, 150001 Harbin, Heilongjiang Province China; 3 The Third Affiliated Hospital of Harbin Medical University, 150040 Harbin, Heilongjiang Province China; 4 Department of Pathology, Harbin Medical University, 150086 Harbin, Heilongjiang Province China

**Keywords:** Senescence, Cancer genetics

## Abstract

The complex interaction between cancer cells and the immune microenvironment is a central regulator of tumor growth and the treatment response. Chemotherapy-induced senescence is accompanied by the senescence-associated secretion phenotype (SASP). However, the mechanisms underlying the regulation of the SASP remain the most poorly understood element of senescence. Here, we show that nuclear erythroid factor 2-like factor 2 (Nrf2), a master antioxidative transcription factor, accumulates upon doxorubicin-induced senescence. This is due to the increased cytoplasmic Inhibitor of Apoptosis Stimulating Protein of P53, iASPP, which binds with Keap1, interrupting Keap1/Nrf2 interaction and promoting Nrf2 stabilization and activation. Activated Nrf2 transactivates a novel target gene of SASP factor, macrophage colony-stimulating factor (M-CSF), which subsequently acts on macrophages and induces polarization from M1 to M2 via a paracrine mechanism. Genetic inhibition of iASPP-Nrf2 suppresses the growth of apoptosis-resistant xenografts, with further analysis revealing that M-CSF/M-CSFR-regulated macrophage polarization is critical for the functional outcomes delineated above. Overall, our data uncover a novel function of iASPP-Nrf2 in skewing the immune microenvironment under treatment-induced senescence. Targeting the iASPP-Nrf2 axis could be a powerful strategy for the implementation of new chemotherapy-based therapeutic opportunities.

## Introduction

Systemic chemotherapy remains the primary treatment for cancer. Drug resistance is a major barrier that limits its effectiveness [1–4]. Accumulating evidence suggests that complex interactions between cancer and immune cells within the tumor microenvironment drive and shape the outcome of cancer [5, 6]. Thus, uncovering the mechanisms underlying communication between cancer and immune cells, in the context of drug treatment, is expected to identify alternative strategies to improve chemotherapy efficiency, leading to optimized clinical outcomes for cancer patients [5, 7–9].

Apoptosis is a major mechanism that contributes to the cytotoxic action of the chemotherapy. However, chemotherapeutic agents can also promote senescence both in vitro and in vivo, which highlights the clinical significance of senescence [10]. An irreversible growth arrest, senescence is considered an intrinsic barrier that limits the expansion of damaged cells [11, 12]. In line with this notion, the occurrence of senescence in pre-malignant lesions or tumors often predicts favorable clinical outcomes [13]. However, senescence is generally accompanied by a secretion phenotype, termed the senescence-associated secretion phenotype (SASP) [11, 13]. The secretome of the SASP consists of a wide range of growth factors, proteases, chemokines, and cytokines, which vary in a cell context-dependent manner. SASP factors affect tumorigenesis or drug responses by influencing tumor cells themselves or the tumor microenvironment via autocrine and/or paracrine mechanisms; however, the functional outcomes of the SASP remain controversial [14–16]. Some SASP factors are elevated in patients receiving chemotherapy treatment and reinforce senescence (stable cell cycle arrest), while others have been reported to promote cell proliferation and invasion, thus conferring a deleterious effect [17, 18].

It should be noted that immune cells have been shown to remove senescent cells in the aging process or in tumor tissues [19–21]. Cancer cells utilize the SASP to induce an antitumor immune response by recruiting immune cells or achieve immune escape by modulating immune cell activities [14]. It is generally accepted that macrophages are the most abundant immune cells within solid tumors, where they can shift to diverse functional phenotypes after infiltration according to local environmental cues [22, 23]. Instead of activating immunity, tumor-associated macrophages (TAMs), which have M2 features, imitate the tissue repair process, suppressing the immune clearance of cancer cells [24]. It has been shown that senescent cells can promote macrophage senescence and induce immunosuppressive M2 macrophage polarization [25]. However, how chemotherapy-induced senescent tumor cells direct immune cells from an active to suppressive state, thus benefiting tumor growth and promoting drug resistance, remains unclear, as is whether oncogenes are involved in modulating this process by changing SASP profiling. Such questions need to be addressed.

Nuclear erythroid factor 2-like factor 2 (Nrf2) is a master regulator of the response to oxidative stress. Under unstressed conditions, it binds with its dominant inhibitor Keap1 in the cytoplasm and is subjected to Keap1-mediated proteasome degradation [26]. Under oxidative stress, Nrf2 is able to escape Keap1 binding, leading to its nuclear translocation and the subsequent expression of antioxidative targets [27–29]. The antioxidative activity of Nrf2 has been demonstrated to be essential in both carcinogenesis and drug resistance [30–33], and studies have also shown that Nrf2 can directly inhibit the transcription of inflammatory cytokines of immune cells [34]. Although oxidative stress is frequently linked to the occurrence of inflammation, whether the expression of Nrf2 in cancer cells influences cancer immunity non-autonomously remains largely unknown.

Here, we show that Nrf2 accumulates after senescence is triggered due to increased expression of an oncogene iASPP (Inhibitor of Apoptosis Stimulating Protein of P53) in the cytoplasm. We go on to identify a novel target of Nrf2 in such a context, macrophage colony-stimulating factor (M-CSF). M-CSF, also known as CSF-1, is a key regulator of macrophage differentiation that acts through M-CSF receptor (M-CSFR) on macrophage. Activation of iASPP-Nrf2-M-CSF induces M2 macrophage polarization non-autonomously. Such functions of iASPP-Nrf2 contribute to chemoresistance both in vitro and in vivo. These data provide evidence for how oncogenes influence senescence and shape the microenvironment to fuel tumor growth and promote chemoresistance, and also suggest that iASPP-Nrf2 is a promising target for the sensitization of drug responses by multiple mechanisms, in addition to well-established cell-autonomous mechanisms.

## Results

### Nrf2 is activated by cytoplasmic iASPP during chemotherapy-induced senescence

**Fig. 1:**
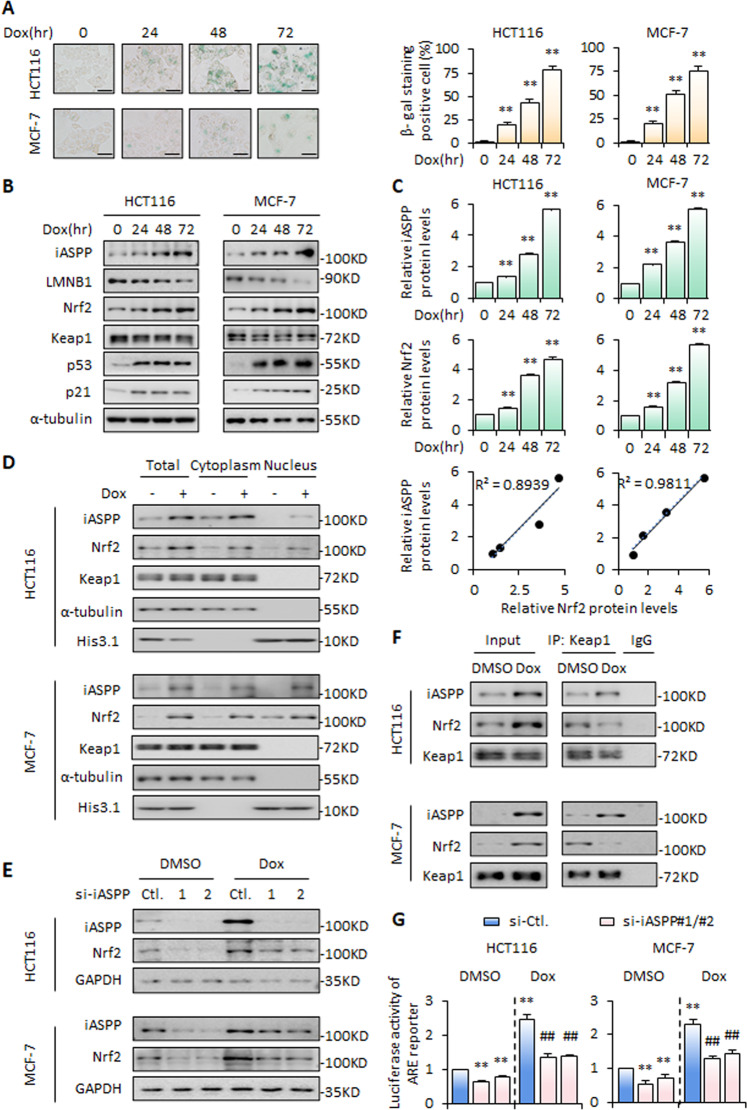
iASPP is required for senescence-induced Nrf2 activation. **A** SA-β-gal staining was performed after triggering senescence in HCT116 and MCF-7 cells. Representative images were presented (left) and the quantification of SA-β-gal staining-positive cells was shown as a bar graph (right). Scale bar = 10 μm. **B**, **C** Expression of iASPP, LMNB1, Nrf2, Keap1, p53, and p21 was determined by western blot in HCT116 and MCF-7 cells with the indicated treatments. Representative blots were presented and α-tubulin was used as an internal control (**B**). The quantification and association of iASPP and Nrf2 protein levels in HCT116 and MCF-7 cells were shown in dot blot (**C**). **D** Distribution of iASPP, Nrf2, and Keap1 in the nucleus and cytoplasm of HCT116 and MCF-7 cells under cellular senescence. **E** The protein expression levels of iASPP and Nrf2 were determined by western blot in HCT116 and MCF-7 cells after the indicated treatments. Representative blots were represented and GAPDH was used as an internal control. **F** The interaction of iASPP, Nrf2, and Keap1 was determined by immunoprecipitation (IP) assay of HCT116 and MCF-7 cells. **G** The luciferase activity of the ARE luciferase reporter in HCT116 and MCF-7 cells were determined after the indicated treatments. Quantitative data are presented as a bar graph. Values are mean ± SD from three independent experiments; **P < 0.01, compared with DMSO (**A**, **C**, **G**); ^##^*P* < 0.01, compared with Dox-treated control (**G**).

To explore the roles of iASPP-Nrf2 in senescence, we first established chemotherapy-induced senescence models [35]. As shown, time-dependent increases of β-Galactosidase (β-gal) activity, p53 and p21 expression, and decreases of lamin B1 (LMNB1) expression were detected in both HCT116 and MCF-7 cells after pulse exposure to a low dose of the chemotherapeutic drug doxorubicin (Dox, 1 μg/mL) for 2 h, following culture in fresh medium for an additional number of days (Fig. 1A, B). BrdU incorporation assay and cell cycle analysis further showed that cell proliferation rates were largely diminished and cells were arrested at G1 phase after triggering senescence (Fig. S1A, B). In line with previous reports, iASPP was found to be increased in a time-dependent manner (Fig. 1B, C) and the increased iASPP was distributed in both the cytoplasm and nucleus in senescent cells (Fig. 1D). Intriguingly, increased Nrf2 expression accompanied the enhanced expression of iASPP after senescence was triggered (Fig. 1B, C). Small interfering RNA (siRNA) that specifically targeted iASPP inhibited senescence-induced iASPP expression and also diminished the expression of Nrf2 (Fig. 1E). Keap1 was predominately localized in the cytoplasm in control and senescent cells (Fig. 1D and Fig. S1C). Nrf2 was predominately localized in the nucleus (Fig. 1D and Fig. S1C). Binding between iASPP and Keap1 was detected under basal conditions and their interaction was found to be increased in senescent cells (Fig. 1F), while the interaction between Nrf2 and Keap1 was correspondingly decreased (Fig. 1F). The transcriptional activity of Nrf2, as indicated by antioxidant response element (ARE) reporter activity, was increased in Dox-treated cells, while iASPP knockdown (KD) abolished Dox-induced ARE activity (Fig. 1G). These data are in agreement with our previously proposed model that iASPP promotes Nrf2 activity by blocking Keap1-Nrf2 interaction and inhibiting Keap1-mediated Nrf2 degradation [36]. Collectively, senescence induces Nrf2 transcriptional activation and this event relies on increased activity of iASPP in the cytoplasm.

### iASPP/Nrf2 axis promotes M-CSF expression in senescent cells

**Fig. 2:**
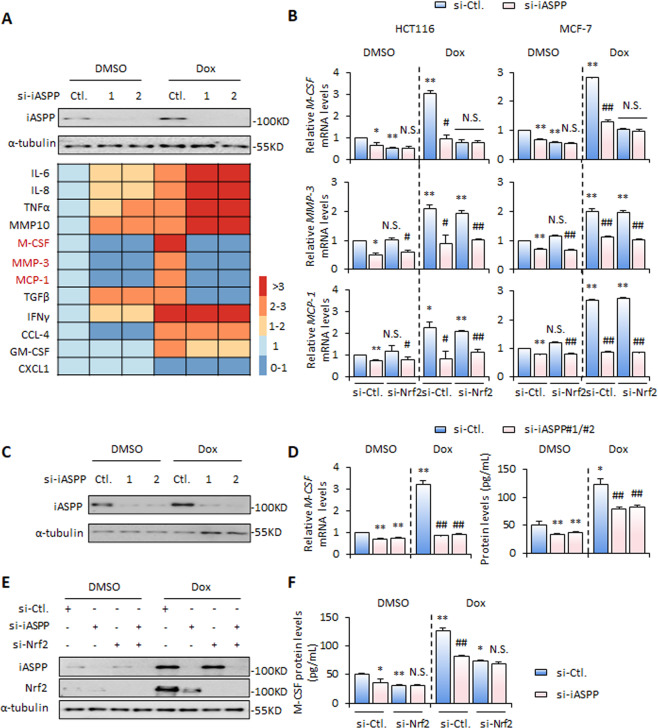
iASPP-Nrf2 axis regulates SASP. **A** mRNA levels of the key SASP factors, *IL-6*, *IL-8*, *TNF-α (Tumour Necrosis Factor-α)*, *MMP10 (matrix metalloproteinase 10)*, *M-CSF*, *MMP3*, and *MCP-1 (Monocyte Chemoattractant Protein-1)*, *TGF-β (Transforming growth factor-β)*, *IFN-γ (Interferon-γ)*, *CCL-4 (C-C Motif Chemokine Ligand)*, and *GM-CSF (Granulocyte-macrophage colony-stimulating factor) CXCL-1 (C-X-C Motif Chemokine Ligand 1)* and *GM-CSF (Granulocyte-macrophage colony-stimulating factor)*, were detected by qRT-PCR before and after triggering senescence in iASPP knockdown (KD) HCT116 cells. **B** mRNA levels of M-CSF, MMP-3 and MCP-1 were detected by qRT-PCR before and after triggering senescence in iASPP and/or Nrf2 KD HCT116 and MCF-7 cells. **C**–**F** mRNA and protein levels of M-CSF were detected by qRT-PCR and ELISA, respectively, after triggering senescence in iASPP and/or Nrf2 KD HCT116 cells (**D**, **F**). KD efficiency of iASPP and/or Nrf2 were confirmed by western blot (**C**, **E**). Values are mean ± SD from three independent experiments; *P < 0.05, **P < 0.01, means compared with DMSO (**B**, **D**, **F**); ^#^*P* < 0.05, ^##^*P* < 0.01, means compared with Dox-treated control (**B**, **D**, **F**); N.S, not significant (**B**).

To explore the role of iASPP-Nrf2 in regulating the SASP, the expression levels of a panel of SASP factors were first examined after inhibition of iASPP expression by si-iASPP. iASPP KD efficiency was confirmed by western blot (Fig. 2A). Quantitative (q)RT-PCR analysis revealed that iASPP KD reinforced senescence-induced *IL-6*, *IL-8*, *TNF-α*, and *MMP10* expression, and abrogated senescence-induced expression of *M-CSF*, *MMP3*, and *MCP-1*. iASPP affected *TGF-β*, *IFN-*, *CCL-4*, and *GM-CSF* differently in a stress-dependent manner, and had no obvious effect on the expression levels of *CXCL-1* in both control and senescent cells (Fig. 2A). These data suggest that iASPP selectively regulates the expression of SASP factors.

We next explored whether Nrf2 contributes to the effect of iASPP on cytokines that it positively regulates under both basal and senescent conditions. The results revealed that Nrf2 KD had no obvious effect on the expression of *MMP3* or *MCP-1* (Fig. 2B); however, *M-CSF* expression was significantly suppressed by si-Nrf2, similarly to the effect mediated by si-iASPP (Fig. 2B). Double treatment with si-Nrf2 and si-iASPP failed to further reduce *M-CSF* levels (Fig. 2B). iASPP-Nrf2-regulated *M-CSF* expression was observed in both senescent HCT116 cells (left panels, Fig. 2B) and MCF-7 cells (right panels, Fig. 2B).

In addition, M-CSF protein expression and secretion were increased in senescent cells. Two independent si-iASPP oligos suppressed iASPP expression (Fig. 2C), which subsequently inhibited senescence-induced *M-CSF* mRNA expression in cancer cells (left, Fig. 2D) and reduced M-CSF protein levels in the culture media (right, Fig. 2D). By contrast, iASPP overexpression increased M-CSF mRNA and protein levels (Fig. S2A–C). Nrf2 KD produced a similar effect as iASPP KD, but no synergistic effect was detected upon double KD (Fig. 2E). In addition, the effect of iASPP-Nrf2 on M-CSF expression was detected under both basal and senescent conditions, and the effect was more pronounced in the latter (Fig. 2F).

iASPP is known to inhibit transcription factors p53 and NF-κB [37, 38]. However, iASPP KD exhibited an obvious effect on M-CSF expression in p53 or NF-κB KD cells (Fig. S2D, E). These data further suggest that iASPP-regulated M-CSF expression is mainly dependent on Nrf2. Thus, the iASPP-Nrf2 axis promotes M-CSF expression and secretion in treatment-induced senescence.

### M-CSF is a novel and direct transcription target of Nrf2

**Fig. 3:**
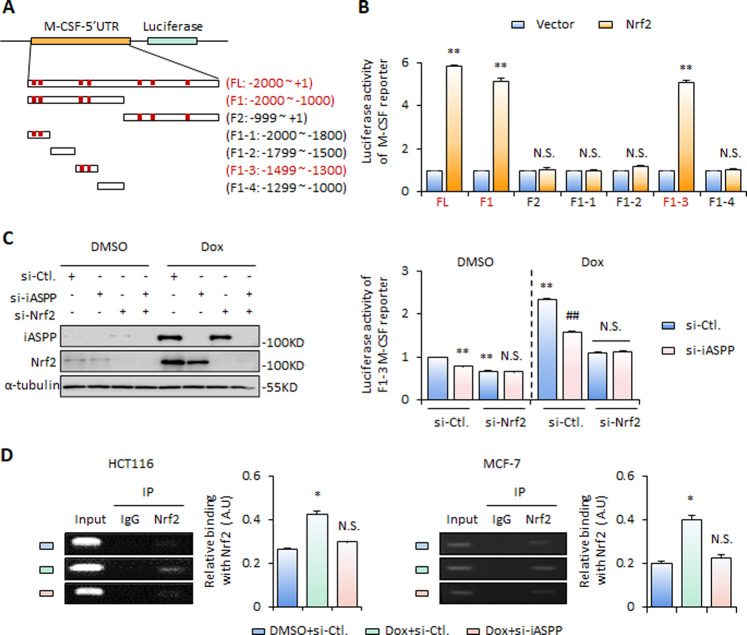
M-CSF is a novel target of Nrf2. **A** Schematic diagram of the M-CSF gene promoter region (−2000 to +1 nt) that contains consensus Nrf2 binding motifs, as predicted by JASPAR software (http://jaspar2016.genereg.net). **B** The activity of M-CSF promoter luciferase reporters was determined by luciferase reporter assay after Nrf2 overexpression (OE). **C** The activity of M-CSF promoter (F1-3) luciferase reporter activity was determined by luciferase assay after triggering senescence in iASPP and/or Nrf2 KD HCT116 cells (**C**). iASPP and Nrf2 KD deficiencies were confirmed by western blot. **D** The interaction between Nrf2 and the M-CSF promoter was analyzed by chromatin IP. Representative images were shown (left). The bands were quantified by Image J and shown in the bar graph (right). Values are mean ± SD from three independent experiments; *P < 0.05, **P < 0.01, compared with DMSO (**B**–**D**); ^##^*P* < 0.01, compared with Dox-treated control (**C**); N.S., not significant (**C**, **D**).

We investigate whether *M-CSF* is a direct transcriptional target of Nrf2. Seven potential Nrf2 binding sites were predicted within a 2000-bp region upstream of the *M-CSF* start sequence by the JASPAR Database (http://jaspar2016.genereg.net) (Fig. 3A). We cloned a full-length sequence and a series of fragmented mutants containing different binding sites upstream of the luciferase reporter (Fig. 3A). The following luciferase reporter assay revealed that two Nrf2 binding sites mapping to fragment (F)1-3 (−1499 to −1300 nt) are required for Nrf2-induced *M-CSF* transcription, because the activities of the luciferase reporter controlled by the full-length (FL), F1(−2000 to −1000 nt) and F1-3 (−1499 to −1300 nt) mutant of the *M-CSF* promoter, but not those controlled by other truncated mutants, responded to Nrf2 overexpression or KD (Fig. 3B, C). iASPP KD similarly reduced F1-3 *M-CSF* luciferase activity and no further reduction was observed when combined with Nrf2 KD (Fig. 3C). The binding between Nrf2 and the *M-CSF* promoter was further validated by chromatin immunoprecipitation using primers spanning the F1-3 *M-CSF* promoter sequence. The Nrf2/*M-CSF* promoter interaction was increased in senescent cells and iASPP KD abolished the senescence-induced Nrf2/*M-CSF* promoter interaction (Fig. 3D).

Increased ROS levels were detected in senescent cells (Fig. S3A) [35, 39]. Inhibition of ROS by ROS scavenger *N*-acetyl-L-cysteine (NAC) abolished senescence-induced oxidative stress (Fig. S3A). Intriguingly, the expression of *M-CSF* appeared to be ROS-independent (Fig. S3B), suggesting that *M-CSF*, a direct target of Nrf2, may be particularly crucial for the anti-inflammatory activity mediated by Nrf2.

### iASPP/Nrf2/M-CSF axis promotes M2 polarization in vitro

**Fig. 4:**
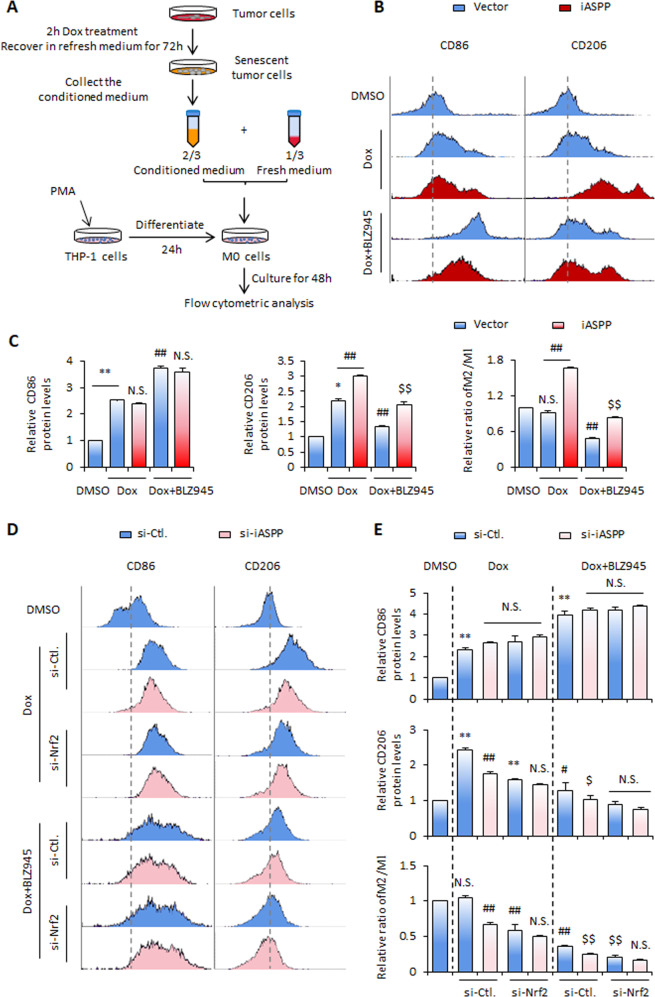
iASPP-Nrf2-M-CSF regulates M2 polarization in vitro. **A** Schematic of the experimental strategies used to trigger macrophage polarization. **B**–**E** Expression levels of CD86 and CD206 were detected by flow cytometry in HCT116 cells after iASPP OE (**B**, **C**), and iASPP and/or Nrf2 KD (**D**, **E**) with and without Dox and/or BLZ945 treatments. The quantification of the ratio of M2/M1 (CD206/CD86) is calculated and shown in bar graphs (**C**, **E**). Values are mean ± SD from three independent experiments; *P < 0.05, **P < 0.01, compared with DMSO (**C**, E); ^#^*P* < 0.05, ^##^*P* < 0.01, compared with Dox -treated control (**C**, **E**); ^$^*P* < 0.05, ^$$^*P* < 0.01, compared with Dox and BLZ945-treated control (**C**, **E**); N.S., not significant (**C**, **E**).

M-CSF is essential in regulating macrophage differentiation via its receptor M-CSFR [40–42]. Although iASPP promoted Dox-induced cell cycle arrest, no obvious effect on cell cycle distribution was detected after Nrf2 KD (Fig. S3C). THP-1 cells were stimulated with Phorbol myristate acetate (PMA) for 48 h to induce them to differentiate into macrophages (M0), as shown in Fig. 4A. The quantities of M2 and M1 macrophages were estimated by assaying two well-established macrophage markers, CD86 for M1 and CD206 for M2, after treatment with conditioned medium (CM) from the indicated cell cultures (Fig. 4A). Both CD86 and CD206 were increased in senescent cells, and their ratio was not changed by the triggering of senescence in the in vitro experimental setting (Fig. 4B, C). However, iASPP overexpression dramatically increased M2 features, as characterized by the upregulation of CD206, but had no effect on CD86, thus resulting in a significant increase of CD206/CD86 (M2/M1) (Fig. 4B, C). Consistently, iASPP overexpression suppressed the expression of M1 cytokines (such as IL-1β, TNF-α, and IL-6) and elevated the expression of M2 markers (such as M-CSF, Arg1, and IL-10) in CM-treated THP1 cells. iASPP KD produced the opposite effect on the levels of these cytokines (Fig. S4A, B). Furthermore, BLZ945, a potent and highly selective small-molecule M-CSFR inhibitor [43], had a dramatic inhibitory effect on the M2 marker CD206. In contrast, levels of M1 marker CD86 were increased by the treatment. BLZ945 led to a decreased M2/M1 ratio, as indicated by the change in the proportions of CD206 relative to CD86 (Fig. 4B, C). Remarkably, iASPP-regulated M2 polarization was compromised by BLZ945 (Fig. 4B, C).

In contrast to the results obtained via iASPP overexpression, inhibition of endogenous iASPP dramatically suppressed CD206 expression, resulting in a significant reduction of CD206/CD86 (M2/M1) ratio. This effect of iASPP KD was alleviated by BLZ945 (Fig. 4D, E). Nrf2 KD had effects similar to those of iASPP KD on macrophage polarization. However, no synergistic effect was detected with their combination (Fig. 4D, E). Collectively, these data support the notion that iASPP/Nrf2/M-CSF drives macrophage M2 polarization.

### Inhibition of the iASPP-Nrf2 axis facilitates apoptosis-resistant xenograft responses to low-dose Dox

**Fig. 5:**
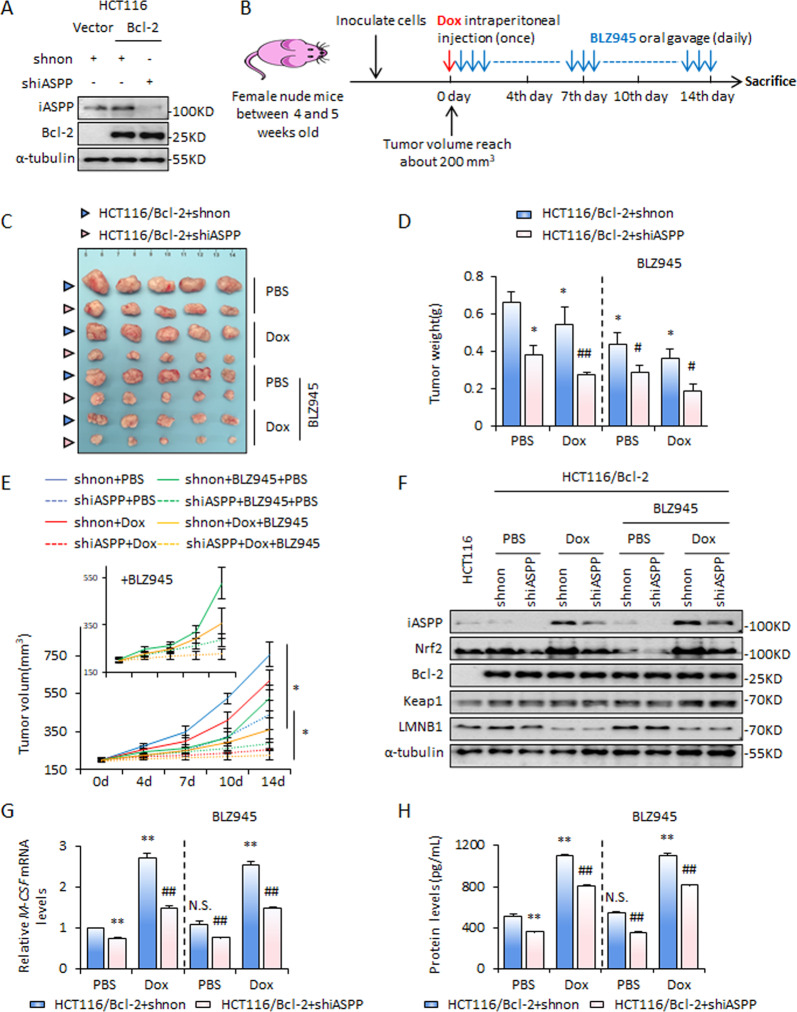
iASPP-Nrf2-M-CSF promotes tumor growth in vivo. **A** The establishment of HCT116/Bcl-2 stable lines was confirmed by the western blot of Bcl-2. **B** Schematic of the Dox and BLZ945 delivery strategy in mice bearing HCT116/Bcl-2 xenografts. **C**–**E** Tumor images (**C**), tumor weights (**D**), and tumor volumes (**E**) of HCT116/Bcl-2 xenografts on the indicated time points after treatments. *N* = 6/group. Values are mean ± SD (**D**, **E**). **F** Expression levels of iASPP, Nrf2, Bcl-2, Keap1, and LMNB1 proteins in the indicated xenografts were determined by western blot. α-tubulin was used as a loading control. **G**, **H** mRNA (**G**) and protein (**H**) levels of M-CSF were determined by qRT-PCR and ELISA in the indicated xenografts. Values are mean ± SEM from three independent experiments; *P < 0.05, **P < 0.01, compared with PBS; ^#^*P* < 0.05, ^##^*P* < 0.01, compared with Dox and/or BLZ945-treated control; N.S, not significant (**G**, **H**).

We next sought to explore the roles of iASPP/Nrf2/M-CSF in regulating tumor growth in vivo. Apoptosis-resistant xenograft (HCT116/Bcl-2) mice models were established as described previously [35] (Fig. 5A). Xenograft-bearing mice were treated with Dox to induce senescence, as indicated in Fig. 5B. The results revealed that iASPP KD HCT116/Bcl-2 xenografts grew relatively slowly compared with the control xenografts (Fig. 5C–E). Dox treatment inhibited tumor growth in both control and iASPP KD xenografts. iASPP KD significantly improved responses of xenografts to Dox (Fig. 5C–E). Western blot analysis confirmed the efficiency of iASPP KD and Bcl-2 overexpression in xenografts (Fig. 5F). Consistent with the results obtained in vitro, Dox treatment led to decreased levels of LMNB1 (Fig. 5F) and Ki67 in xenografts (Fig. S5A). iASPP KD augmented the inhibitory effect of Dox on cell proliferation. iASPP expression increased after Dox treatment, accompanied by an increase in Nrf2, while iASPP KD abolished the effect of Dox on Nrf2 expression (Fig. 5F and Fig. S5B). Similar changes to the expression of M-CSF at both the mRNA and protein levels were detected in the same set of samples (Fig. 5G, H). These data suggest that Dox inhibits tumor growth by inducing cellular senescence and inhibiting the iASPP-Nrf2-M-CSF axis improved treatment outcomes with Dox.

### M-CSF/MCFR signaling contributes to iASPP-Nrf2-mediated tumor growth in vivo

**Fig. 6:**
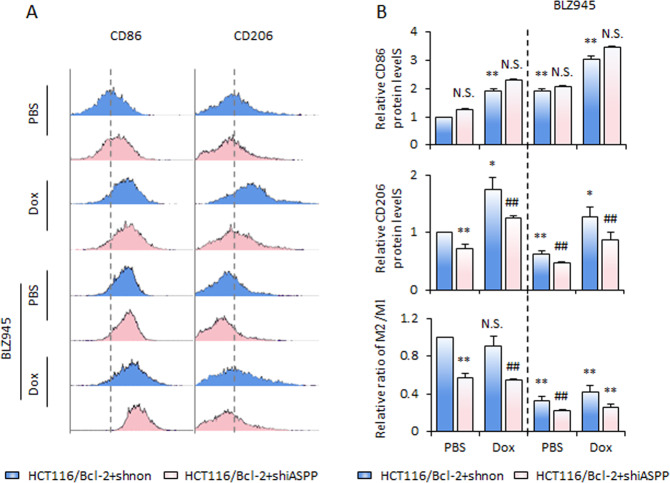
iASPP-Nrf2-M-CSF regulates M2 polarization in vivo. **A**, **B** Expression levels of CD86 and CD206 were detected by flow cytometry in the indicated xenografts. The representative images are shown in **A** and the quantification and the ratio of M2/M1 (CD206/CD86) are shown in **B**. Values are mean ± SD from three independent experiments; *P < 0.05, **P < 0.01, compared with PBS (**B**); ^##^*P* < 0.01, compared with Dox and/or BLZ945-treated control (**B**); N.S., not significant (**B**).

To understand the contribution of macrophage polarization to the effect of iASPP KD in vivo, we compared the effect of iASPP KD in the presence or absence of BLZ945 (Fig. 5B). Inhibition of M-CSF/M-CSFR signaling with BLZ945 had a significant antitumor effect and also sensitized xenografts to respond to Dox (Fig. 5C–E). BLZ945 compromised but did not completely abolish, the antitumor effect of iASPP (Fig. 5C–E). These data suggest that iASPP has both M-CSF-dependent and -independent pro-tumor functions in vivo, which is consistent with the data in Fig. 4 showing that iASPP-Nrf2 regulation of macrophage polarization is largely, though not wholly, dependent on M-CSF. Although BLZ945 had no obvious effect on the expression of M-CSF in HCT116/Bcl-2 xenografts (Fig. 5G, H), it significantly promoted CD86 expression and suppressed CD206, producing a negative effect on M2 polarization, as indicated by the decreased ratio between CD206/CD86 (M2/M1) (Fig. 6A, B). iASPP KD had no obvious effect on CD86, while it elicited a significant effect on CD206 expression, leading to a robust reduction of CD206/CD86 ratio. Furthermore, the effect of iASPP was largely comprised of BLZ945 (Fig. 6A, B). Collectively, these data are consistent with our in vitro findings and indicate that the ASPP-Nrf2-M-CSF axis contributes to M2 polarization, resulting in tumor growth in vivo.

### Expression levels of iASPP/Nrf2/M-CSF in human colon cancer specimens

**Fig. 7:**
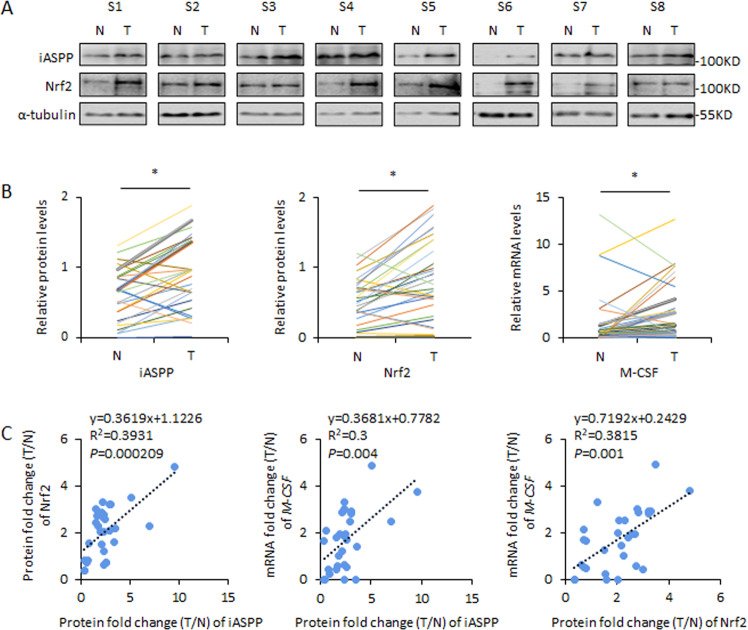
iASPP-Nrf2-M-CSF are associated with each other in vivo in colon cancer tissues. **A**, **B** Representative western blot (**A**) and quantification (**B**) of iASPP and Nrf2 protein levels and M-CSF mRNA levels in 30 pairs of human colorectal cancer samples (T) and paired adjacent normal controls (N). **C** The linear correlation analysis of the fold change of iASPP protein expression vs those of Nrf2 protein expression, the fold change of iASPP protein expression vs those of M-CSF mRNA expression, and the fold change of Nrf2 protein expression vs those of M-CSF mRNA expression. Values are mean ± SD from three independent experiments; *P < 0.05 (**B**).

Given the important roles of iASPP/Nrf2/M-CSF in tumor growth, we further investigated their association in human tissues. As shown, 30 pairs of colon cancer (T) tissues and their paired adjacent normal controls (N) were collected and subjected to western blot (Fig. 7A) and qRT-PCR analysis (Fig. 7B). The results showed that iASPP and Nrf2 protein levels were increased in colon cancers compared with their paired normal controls. The fold change (T/N) of iASPP was proportional to that of Nrf2, suggesting that iASPP overexpression may contribute to the increased Nrf2 expression observed in colon cancers in vivo (Fig. 7B). In addition, M-CSF mRNA levels were higher in colon cancers than in the normal controls (Fig. 7B). Intriguingly, M-CSF mRNA levels were positively associated with the protein levels of iASPP and Nrf2 (Fig. 7C). These findings suggest that the newly identified iASPP/Nrf2/M-CSF axis may indeed be present in vivo in human tissues.

## Discussion

**Fig. 8:**
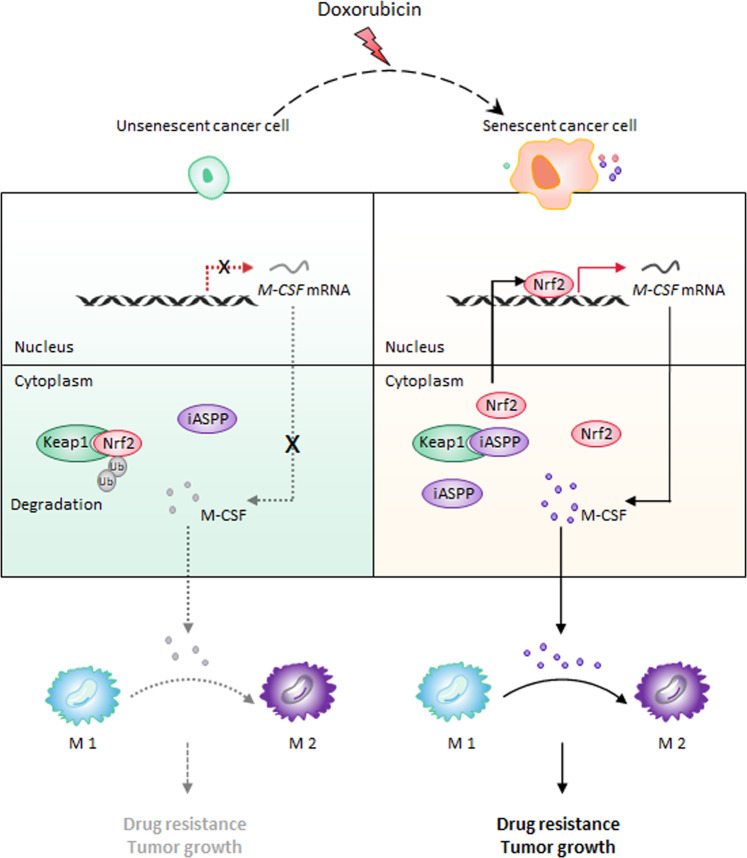
Schematic working model for the role of iASPP-Nrf2-M-CSF in regulating M2 polarization in the context of chemotherapy-induced senescence. The expression of Nrf2 is increased after triggering senescence in cancer cells. This is due to the parallel enhancement of iASPP. Increased iASPP binds with Keap1 in the cytoplasm, and thereby suppresses Keap1-mediated Nrf2 degradation. The accumulated Nrf2 enters into the nucleus, where it binds to the promoter region of M-CSF gene and transactivates M-CSF expression. M-CSF secretes into the microenvironment and contributes to the iASPP-Nrf2-mediated M2 polarization, resulting in tumor growth and resistance to Dox-mediated anti-tumor effects.

Toxic side effects are the dark side of chemotherapy. Scientists have found that low-dose treatment can attenuate such effects by promoting senescence; however, senescence is a complicated process that is accompanied by the SASP, which can either promote cancer immunity or lead to immune evasion [44, 45]. How the opposing effects of the SASP can be distinguished and treatment efficacy improved remains an important issue in cancer research. Here, we reveal a mechanism by which cancer cells reshape the immune microenvironment by inducing iASPP-Nrf2-M-CSF-mediated M2 polarization to attenuate the antitumor effect of Dox (Fig. 8).

iASPP is an oncogene that is overexpressed in multiple types of cancer. Previous evidence has suggested that iASPP mainly acts by inhibiting apoptosis or the cell cycle arrest of cancer cells [35, 36, 46], and whether iASPP regulates cancer and immune cell communication has been beyond our understanding. Here, we have shown that iASPP can promote tumor growth by influencing macrophage differentiation via cancer-secreted SASP factors. Intriguingly, iASPP is involved in the regulation of multiple SASP factors through diverse mechanisms. For example, it inhibits NF-κB-regulated IL-6/8 expression, leading to cell proliferation [35], and activates Nrf2-regulated M-CSF expression, resulting in immune evasion. Despite the complicated nature of the SASP, the functional outcome of iASPP in regulating the SASP is likely to mitigate the antitumor effect of senescence, suggesting that inhibition of iASPP may be a potential treatment approach to selectively reshape SASP profiling and improve treatment efficiency. It should also be noted that iASPP inhibits or promotes the expression of additional SASP factors, such as MMP10 and TNF-α, at least at the mRNA level. Whether these downstream factors also contribute to the iASPP-mediated interconnectivity between cancer cells and the microenvironment warrants further investigation. Furthermore, heart and fibroblast cells isolated from iASPP-defective mutant mice or human patients are more sensitive to lipopolysaccharide (LPS)-induced inflammation [47]. It has been previously suggested that this activity of iASPP is mainly attributed to altered NF-κB activity, while our study suggests that the contribution of Nrf2 also needs to be considered in such a context. Moreover, cell senescence is a very complex process that involves at least two arms: cell cycle arrest and the SASP [48–51]. iASPP regulates both effects, leading to the retardation of cell cycle arrest by suppressing p53 and NF-κB, and impairing immune surveillance by activating Nrf2, which suggests that iASPP may be a central hub that connects the key transcription factors (p53, NF-κB, and Nrf2) involved in senescence. Therefore, iASPP-targeted therapy is plausible to improve the anti-tumor effect of chemotherapy-induced senescence. However, it has been recently reported that iASPP also plays a role to enhance the self-renewal ability of hematopoietic stem cells [52]. Thus, cancer-specific strategies need to be developed to avoid the possible hematopoietic toxicity.

Oxidative stress and inflammation are interconnected, thus it is not surprising that Nrf2, a well-known master antioxidative regulator, has been reported to contribute to anti-inflammation [53, 54]; however, it has long been believed that anti-inflammation is merely a consequence of ROS elimination [34]. The underlying mechanisms of Nrf2-mediated anti-inflammation have long been ignored. Here, we identify M-CSF to be a novel inflammation-related target of Nrf2. Although Nrf2-regulated M-CSF expression is dependent on the ARE sequence that maps to its promoter region, the activation of Nrf2-regulated M-CSF is ROS-independent, suggesting that Nrf2 utilizes similar mechanisms to mitigate oxidative and inflammatory stress by transactivating different targets in cancer cells. Recently, Kobayashi et al. reported that Nrf2 suppresses LPS-induced transcriptional upregulation of proinflammatory cytokines, including IL-6 and IL-1β, in macrophages [34]. Instead of binding to the ARE regions of its target genes, this activity of Nrf2 is ARE sequence- and oxidative stress-independent. It is likely that different cells activate Nrf2-mediated anti-inflammatory effects via diverse mechanisms. In addition, we found that Nrf2 has no obvious effect on the expression of IL-6 in senescent cancer cells [35], further suggesting that Nrf2-regulated cytokine expression is cell context-dependent. Thus, Nrf2 utilizes different mechanisms to fulfill its anti-inflammatory roles, inhibiting cytokine expression in macrophages and promoting M-CSF expression in cancer cells, both of which inhibit macrophage activity autonomously or non-autonomously in a ROS-independent manner.

M-CSF is essential for macrophage differentiation, which is frequently overexpressed in tumors, and increased M-CSFR levels are associated with poor prognosis in patients with various cancers [40, 41, 55, 56]. Interestingly, M-CSF is among the most dramatically changed genes after iASPP-Nrf2 KD, and our data also show that iASPP regulates macrophage polarization mainly by inhibiting senescence-induced M-CSF expression and secretion in cancer cells. We recently reported a novel mechanism of iASPP inhibition of drug-induced apoptosis via the activation of Nrf2-mediated antioxidative signaling [36]. Here, we provide the first evidence that iASPP can modulate Nrf2’s activity and elicit a non-autonomous effect that stimulates macrophage polarization by inducing M-CSF expression. Recently, “One-two-punch” therapy strategies have attracted great attention, including those inducing tumor cell senescence followed by selective clearance of senescent cells [57]. Unveiling the multiple facets of iASPP function in therapy-induced senescence may provide important insights into developing “one-two punch” cancer therapy. In addition, since senescence can, paradoxically, promote tumor relapse, and drug resistance [58], the long-term effect of iASPP-Nrf2 on therapy-induced senescence warrants further investigation. Furthermore, the positive association between iASPP/Nrf2 and M-CSF in colon cancer tissues suggests that activation of the iASPP/Nrf2 axis may contribute to the increased expression of M-CSF in vivo, at least in colon cancers.

## Conclusions

Collectively, our studies have identified a novel biological function for the classic antioxidative Nrf2 and the antiapoptotic iASPP in the SASP. iASPP-Nrf2 inhibition could be a powerful strategy to restore senescence and may represent a new target to shape the SASP and implement chemotherapy-based therapeutic opportunities.

## Materials and methods

### Colon cancer patient samples

Thirty human colorectal cancer tissues and their corresponding adjacent normal controls were collected from the Third Affiliated Hospital of Harbin Medical University, China. Written informed consent was obtained from all patients. All samples were obtained immediately after the operation and stored in liquid nitrogen. The total proteins and RNAs were extracted and then analyzed to western blot and qRT-PCR. The study has been approved by the Research Ethics Committee of Harbin Medical University, China.

### Cell lines and treatments

The human colorectal cancer cell line HCT116 (ATCC), the human breast cancer cell lines MCF-7 (ATCC), and the human monocyte cell lines THP-1 were maintained RPMI-1640 medium (Gibco) supplemented with 10% (v/v) fetal bovine serum (Biological Industries). All cells were grown in a humidified incubator (Thermo Scientific) containing 5% CO_2_ at 37 °C, and had not been passaged for 3 months before the experiment. The cell line was routinely tested to exclude mycoplasma contamination and characterized by the use of short tandem repeat markers by the Genetic Testing Biotechnology Company (Suzhou, China). All siRNA oligos and plasmids were introduced into cells by Lipofectamine 2000 (Invitrogen) according to the manufacturer’s instruction. Use 1 μg/mL doxorubicin (Selleck Chemicals) and DMSO as a control to perform the specified analysis on the cells. For drug treatments, HCT116 and MCF-7 cells were pulse-exposed to doxorubicin for 2 h, and then replaced with fresh medium, and the cells were cultured for additional several days. THP-1 cells were differentiated into macrophages by treatment with 320 nM of phorbol 12-myristate 13-acetate (PMA) for 24 h, and then recovered 2/3 of conditioned medium and 1/3 of fresh medium. THP-1 cells were treated with 500 nM BLZ945 (Selleck Chemicals) for 48 h to inhibit M-CSF/M-CSFR axis.

### In vivo xenograft mouse study

HCT116 cells were infected with PLKO.1-iASPP (short hairpin RNA, shRNAiASPP) lentivirus to knock down iASPP expression (HCT116/shiASPP), and blank lentivirus was used as a control (HCT116/shnon). Both stable cell lines were infected with Bcl-2 lentivirus (Bcl-2) and its blank lentivirus control (vector) to avoid the effects of apoptosis induced by Doxorubicin. HCT116/Bcl-2/shnon and HCT116/Bcl-2/shiASPP single clones were selected and Bcl-2 overexpression and iASPP KD were confirmed by WB. The female nude mice between 4 and 5 weeks old were purchased from Beijing HFK Bioscience Co., Ltd. In all, 1 × 10^7^ pairs of cells were subcutaneously inoculated on both sides of the back abdomen of the same female nude mice. The body weights of the mice, the tumor size, and the tumor volumes were measured every week and calculated as length × width^2^ × 0.5. When the tumor volume reached about 200 mm^3^, the mice bearing HCT116/Bcl-2/shnon and HCT116/Bcl-2/shiASPP were randomly divided into 4 groups, respectively (*n* = 5/group). The mice were treated with Dox (10 mg/kg, intraperitoneal) to establish a senescence model, combined with or without M-CSFR inhibitor BLZ945 (200 mg/kg, oral gavage, once a day). After 2 weeks of drug treatments, the mice were anesthetized and eliminated. The tumor was carefully removed, photographed, and weighed. All animal procedures were performed according to protocols approved by the Rules for Animal Experiments published by the Chinese Government (Beijing, China) and approved by the Research Ethics Committee of Harbin Institute of Technology, China.

### Western blot

The different samples of cells were lysed in urea buffer containing 2 M Thiourea, 4%CHAPS, 40 mM Tris-Base, 40 mM DTT, 2% Pharmalyte and sonicated to shear DNA. Protein expression was detected by ECL and visualized by Image studio system (ECL, LI-COR, Lincoln, Georgia, USA). Image J software (National Institutes of Health, Bethesda, MD, USA) was used to quantify protein expression. The sources and dilution ratio of the primary antibodies are shown as follows: anti-iASPP (Sigma, #A4605, 1:2000), anti-LMNB1 (Proteintech, #12987-1-AP, 1:2000), anti-Keap1 (Proteintech, #10503-2-AP, 1:2000), anti-Bcl2 (Proteintech, #12489-1-AP, 1:2000), anti-GAPDH (Proteintech, #10494-1-AP, 1:2000), anti-α-tubulin (Proteintech, #11224-1-AP, 1:2000), anti-Histone-H3.1 (Proteintech, #17168-1-AP, 1:2000), anti-p53 (Proteintech, #10442-1-AP, 1:2000), anti-p21 (Proteintech, #10355-1-AP, 1:500) and anti-Nrf2 (Proteintech, #16396-1-AP, 1:1000).

### RNA extraction and quantitative (q)RT-PCR

According to the experimental procedure provided by the manufacturer, total RNA isolated with Trizol (Invitrogen) was reverse transcribed with GoScriptTM Reverse Transcription System (Promega), and qRT-PCR was performed by using SYBR Premix Ex Tag II (TaKaRa). The gene expression level relative to the 18s rRNA control was calculated by the 2^−ΔΔct^ method.

### Enzyme-linked immunosorbent assay

The AuthentiKine^TM^ human M-CSF ELISA kit was used to measure the concentration of M-CSF in the cell culture medium by the ELISA method according to the manufacturer’s instructions (Proteintech). The absorbance of the sample was measured at 450 and 630 nm. Each experiment was repeated 3 times.

### Luciferase reporter assay

M-CSF promoter regions at (−2000 to +1 nt, FL) were obtained by PCR using genomic DNA obtained from 293T cells as a template and then cloned into pGL3-basic luciferase reporter plasmid. The luciferase reporter controlled by truncated mutants, including (−2000 to −1000 nt, F1), (−999 to +1 nt, F2), (−2000 to −1800 nt, F1-1), (−1799 to −1500 nt, F1-2), (−1499 to −1300 nt, F1-3), and (−1299 to −1000 nt, F1-4) were obtained by sub-colony. The same amount M-CSF promoter-luciferase reporter plasmid together with expression plasmids for Nrf2 or iASPP/si-Nrf2 or si-iASPP were transfected into the indicated cancer cells. Each transfection contained the same amount of Renilla, which was used for standardization control. 48–72 h after transfection, the cells were treated with Dox or DMSO control for 2 h, recovered with fresh medium, and cultured for an additional 72 h. Finally, the luciferase activity was detected using a luciferase assay system (Promega) by following the manufacturer’s introduction. The relative luciferase activity is normalized with Renilla luciferase activity.

### BrdU incorporation assay

BrdU incorporation assay was carried out by following the protocol provided by Cell Signaling Technology. Briefly, BrdU diluted at a final concentration of 0.03 mg/mL with fresh DMEM medium was applied onto the cells grown on slices. Cells were then incubated with 1.5 M HCl followed by 5 min fixation in 70% cold ethanol. After blocking with 3% BSA, cells were incubated with anti-BrdU antibody (CST, #5292 S, 1:1000 dilution) overnight, followed by another round of incubation with fluorescent secondary antibody (Thermo, #A28181) at room temperature for 1 h. The nuclei were visualized by DAPI staining. The representative images were captured by a Zeiss LSM510 confocal microscope (Carl Zeiss, Heidelberg, Germany).

### ROS measurement

Incubate the tumor cells from which the culture medium has been removed with 10 μM dichlorodihydrofluorescein diacetate (DCFH-DA, Sigma) in a 37 °C CO_2_ incubator for 30 min. The cells were then washed with PBS and digested with trypsin. Trypsinized cells were resuspended in PBS solution and kept single-cell suspension on ice until rapid analysis by FACS. The data is reported as the fold change of the average fluorescence intensity, normalized to the fluorescence intensity of untreated control cells.

### Chromatin immunoprecipitation

The cells were incubated with formaldehyde to produce protein-DNA cross-linked complexes, which were then purified and sheared by sonication. The chromatin was divided evenly into two groups for further IP reaction with anti-Nrf2 antibody or IgG control. The immunoprecipitates were pelleted by centrifugation and then incubated at 65 °C to reverse protein-DNA crosslinks. The DNA was extracted with an Axygen product purification kit. The same amount of precipitated DNA fragments was subjected to PCR analysis at 58 °C for 33 cycles by 2 × GoldStar Best MasterMix (Cwbiotech) and visualized by running 1% agarose gel.

### Immunoprecipitation (IP)

Cells were lysed in NETN buffer (50 mM Tris-HCl [pH = 8.0], 150 mM NaCl, 1% NP-40, 1 mM EDTA), with Proteinase Inhibitor Cocktail (MedChemExpress, #HY-K0010) added before use. After the resulting lysate was precleaned by protein G sepharose beads 4 Fast Flow (GE Healthcare, #17061802), specific antibodies or control IgG was added to the supernatant, which was incubated with FBS blocked beads on a rotating wheel at 4 °C overnight. Beads with the bound immunoprecipitates were collected following four washes with cold NETN and then the immunoprecipitates were analyzed by WB assay.

### Immunohistochemistry

The tumor tissue sections were subjected to antigen retrieval by boiling in 0.01 mol/L citrate buffer for 5 min. After cooling down to room temperature, the tissue sections were incubated with anti-ki67 (Proteintech, #27309-1-AP, 1:5000) antibody at 4 °C overnight. After that, the tissue sections were washed three times with PBS. The second antibody was then added followed by 1 h incubation at room temperature. Detection was carried out by the REAL EnVision detection system (Dako) with diaminobenzidine peroxidase serving as chromogen after PBS washes. The tissue sections were observed under the microscope and the images were captured for further intensity analysis.

### SA-β-Gal staining

SA-β-gal staining is the most classic assay to detect cellular senescence. Senescent cancer cells were washed with PBS three times, then fixed with 0.2% glutaraldehyde and 37% formaldehyde solution for 5 min. Added β-gal staining solution after PBS rinsing, Its composition is 1 mg/mL 5-bromo-4-chloro-3-inolyl-β-D- galactoside (X-gal) in staining solution (dimethyformamide (20 mg/mL stock), 40 mM citric acid/sodium phosphate, pH = 6.0, 5 mM potassium ferrocyanide, 5 mM potassium ferricyanide, 150 mM NaCl and 2 mM MgCl_2_. The cells were incubated in a 37° C incubator without CO_2_ for 12–14 hr. After incubation, the cells were washed three times with PBS and photographed. The number of cells stained positive was counted.

### Flow cytometric analysis

The in vitro cultured THP-1 macrophage or macrophage collected from *the* in vivo xenografts were treated with 4% paraformaldehyde for 10 min, and then washed by PBS three times. The cells were then incubated with anti-CD86 (Abcam, #ab53004, 1:200) antibody and anti-CD206 (Abcam, #ab64693, 1:200) antibodies, respectively, at room temperature for 1 h. After three times washed in PBS, cells were incubated with the secondary antibody of the corresponding species for an additional 1 h. The macrophages were washed three times with PBS and resuspended in 1 ml of PBS for flow cytometric analysis.

### Cell cycle analysis

The cells were washed with PBS, detached with 0.25% trypsin, and fixed with 75% ethanol overnight. After treatment with 1 mg/mL RNase A (Sigma) at 37 °C for 30 min, resuspended in 0.5 mL of PBS and stained with propidium iodide in the dark for 30 min. Then the cell cycle distribution was detected by flow cytometry.

### Cell fraction

Cytoplasm lysis buffer (10 mM HEPES pH = 7.9, 10 mM KCl, 1.5 mM MgCl_2_, 0.5 mM β-mercaptoethanol) was applied to cells, followed by moderate vortex for 15 s and 15–20 min incubation on ice. Additional 5 μL 10% NP-40 (Amersco) was then added to the mixture followed by another round of vortex and incubation. The cytoplasm fraction was obtained by collecting supernatant after centrifugation at 16,000 × *g* for 10 min at 4 °C. The resulting pellet was lysed in the nuclear fraction buffer (10 mM HEPES pH = 7.6, 1 mM DTT, 7.5 mM MgCl_2_, 0.2 mM EDTA, 0.3 M NaCl, 1 M UREA, 1% NP-40). The supernatant was collected as the nuclear fraction by centrifugation at 16,000 × *g* for 10 min at 4 °C.

### Statistical analysis

Statistical analysis was done by the GraphPad software, version 5. Correlation analysis was conducted by SPSS software. Data are presented as the means ± standard error of the means (SEM) or standard deviation (SD). Student’s *t* test was applied to assess the statistical significance. Any *P* value of <0.05 is regarded as statistically significant.

## Supplementary information

Original western blots

aj-checklist

Supplementary figure

## Data Availability

Data used or analyzed during the current study are available from the corresponding author on reasonable request.
