# Supplementary figures and images for: Blocking iASPP/Nrf2/M-CSF axis improves anti-cancer effect of chemotherapy-induced senescence by attenuating M2 polarization
Source: Cell Death Dis. 2022 Feb 21;13(2):166. doi: 10.1038/s41419-022-04611-4 (PMC8861031; doi:10.1038/s41419-022-04611-4)

Supplementary Material

(Western Blots)


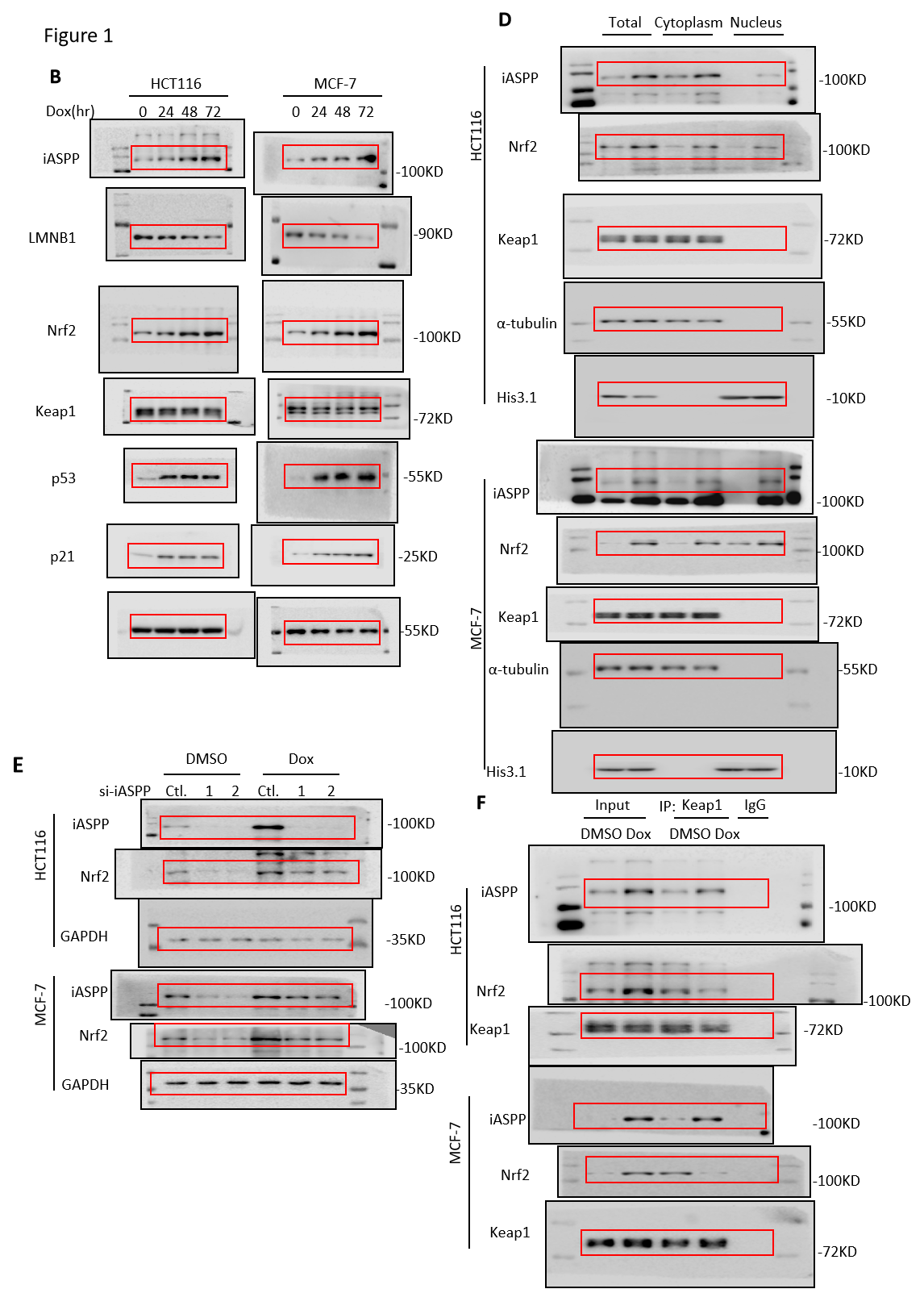


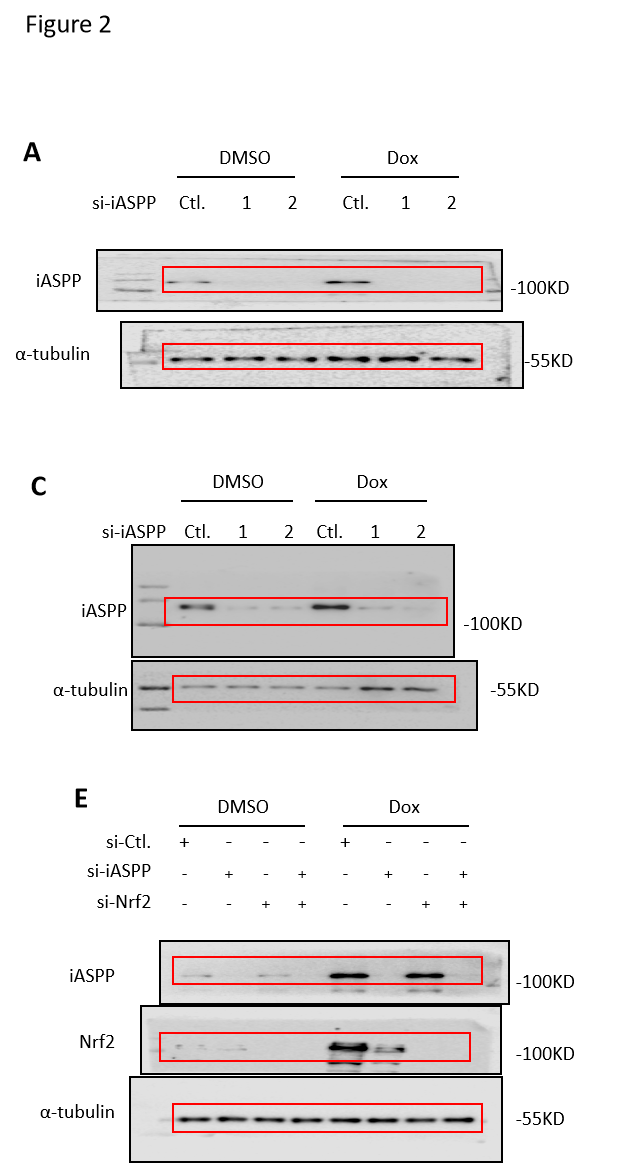


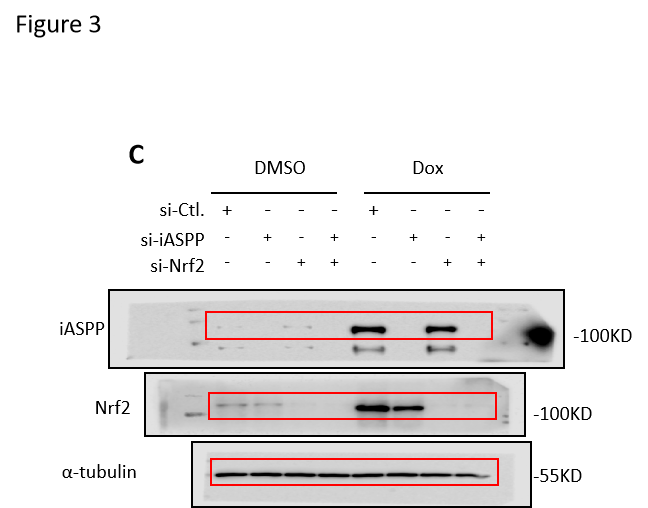


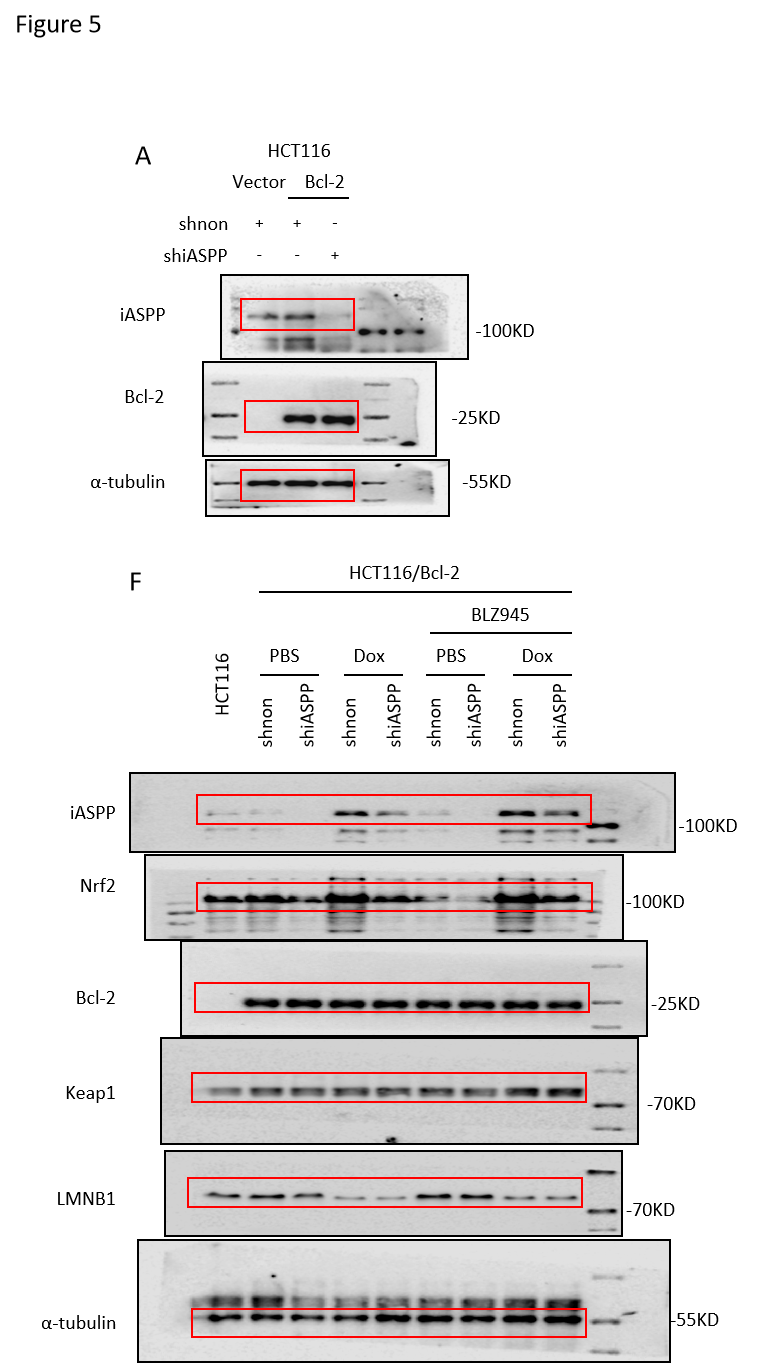


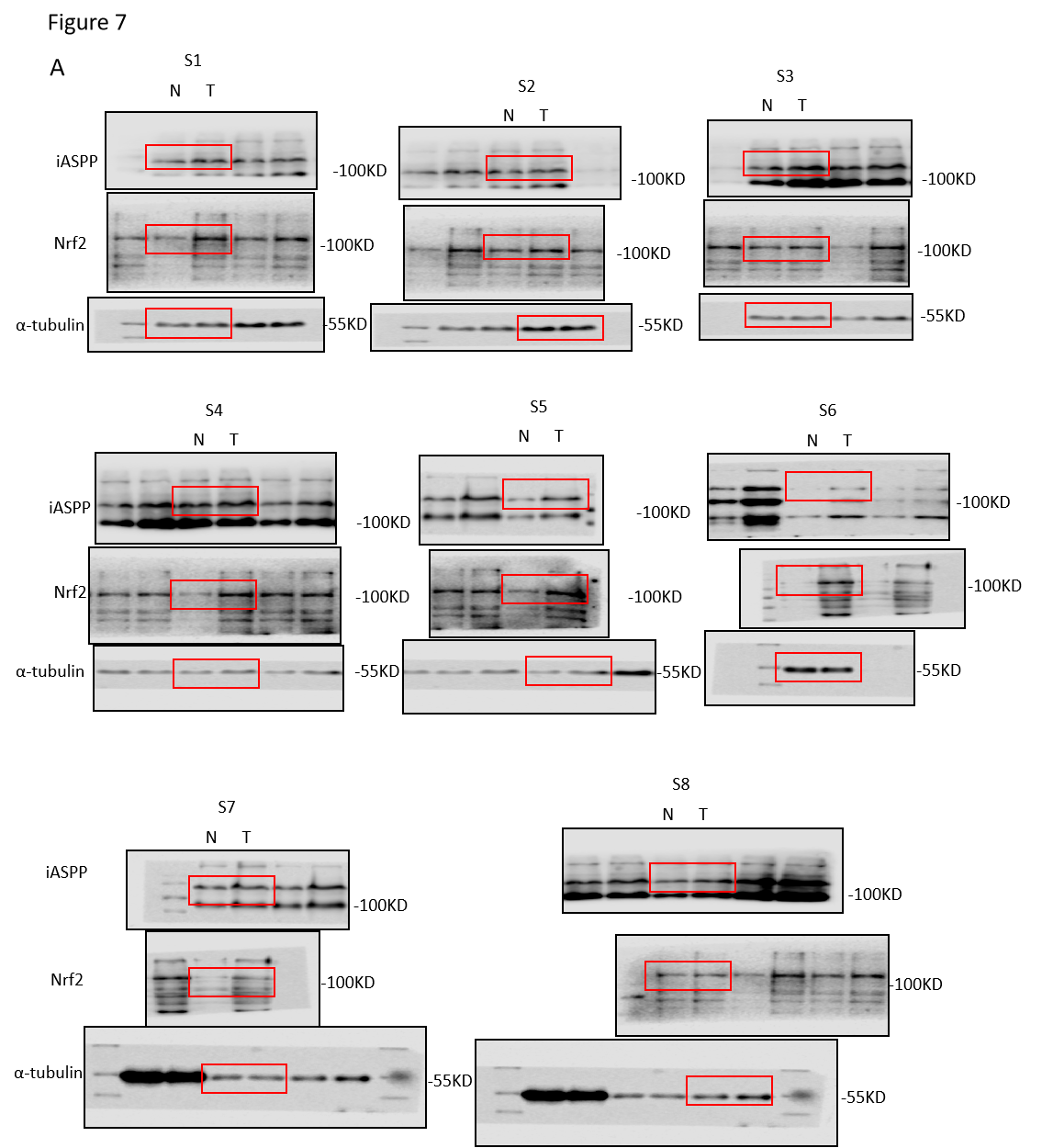


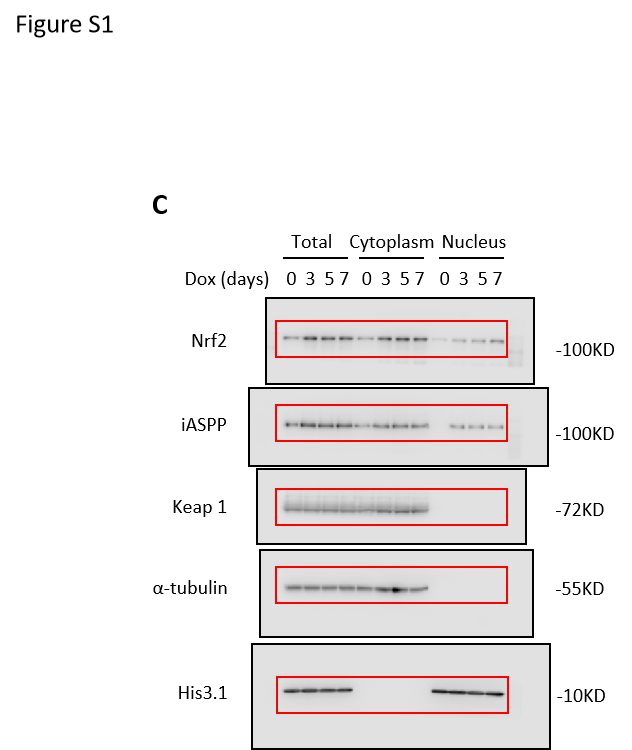


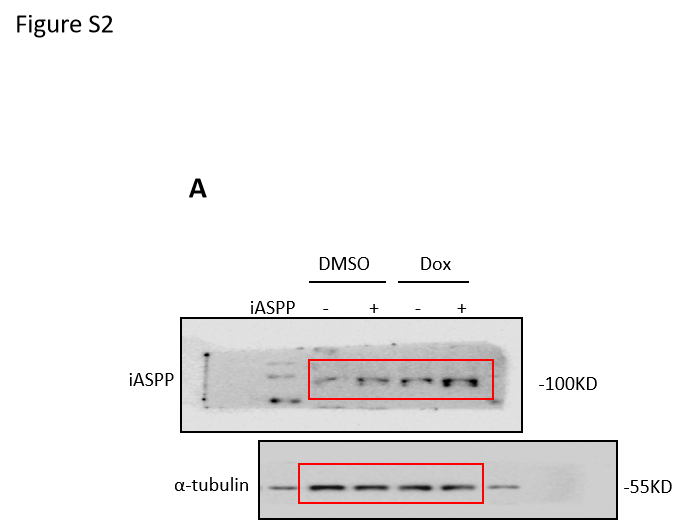

Supplement: Supplementary file 1 — Original western blots [file 41419_2022_4611_MOESM1_ESM.docx]
