# Supplementary material for: Blocking iASPP/Nrf2/M-CSF axis improves anti-cancer effect of chemotherapy-induced senescence by attenuating M2 polarization
Source: Cell Death Dis. 2022 Feb 21;13(2):166. doi: 10.1038/s41419-022-04611-4 (PMC8861031; doi:10.1038/s41419-022-04611-4)
Supplement: Supplementary file 3 — Supplementary figure [file 41419_2022_4611_MOESM3_ESM.docx]

**Blocking iASPP/Nrf2/M-CSF axis improves anti-cancer effect of chemotherapy-induced senescence by attenuating M2 polarization**

Hao Liu^1,2,#^, Dong Zhao^2,#^, Huayi Li^2^, Wenxin Zhang^2^, Qingyu Lin^2^, Xingwen Wang^2^, Shanliang Zheng^2^, Lei Zhang^3^, Li Li^4^, Shaoshan Hu^1,^*, Ying Hu^2,^*

1 Department of Neurosurgery, Emergency Medicine Center, Zhejiang Provincial People's Hospital, Affiliated to Hangzhou Medical College, Hangzhou, Zhejiang, China, 310000

2 School of Life Science and Technology, Harbin Institute of Technology, Harbin, Heilongjiang Province, China, 150001

3 The third affiliated hospital of Harbin Medical University, Heilongjiang Province, China, 150040

4 Department of Pathology, Harbin Medical University, Heilongjiang Province, China, 150086

* To whom correspondence should be addressed. Tel: 0086-86403826; Fax: +86-451-86403826; Email: [huying@hit.edu.cn](mailto:huying@hit.edu.cn), shaoshanhu421@163.com

^#^ These two authors contribute equally to the work.

**Key words:** iASPP, Nrf2, M-CSF, macrophage polarization, resistance

**Supplementary Figures**


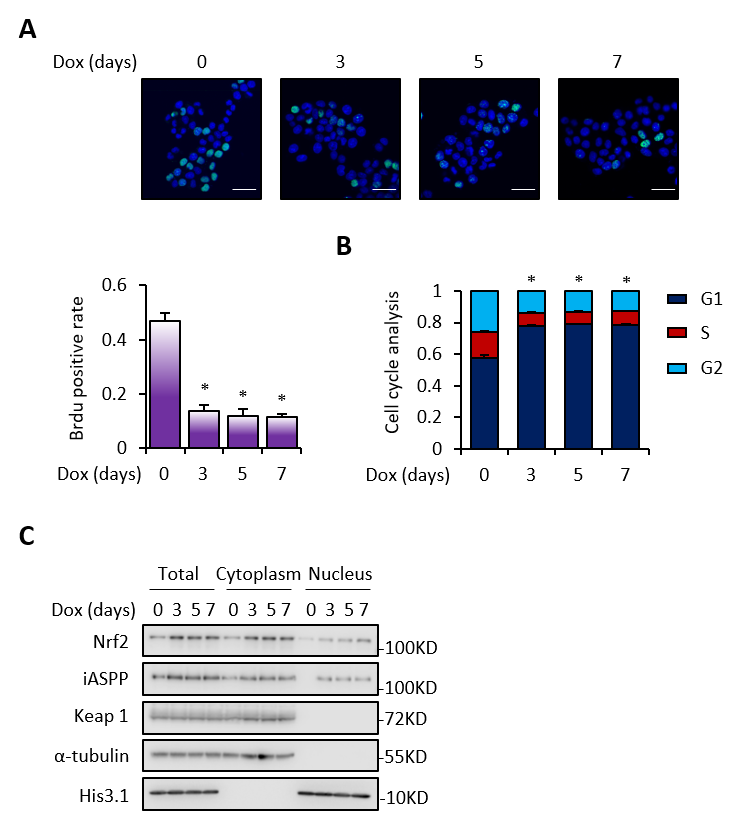


**Figure S1 Chemotherapy-induced senescence promotes Nrf2 expression**

(A) The proliferation rates were determined by BrdU incorporation assay, 3, 5 and 7 days after triggering senescence in HCT 116 cells. Quantitative data are presented as a bar graph. Bar=10μm. (B) The cell cycle distribution was detected by flow cytometry following PI staining in HCT116 cells under the control and senescent conditions. (C) Distribution of iASPP, Nrf2 and Keap1 in the nucleus and cytoplasm of HCT116 cells under the conditions of chemotherapy-induced senescence. Values are mean ± SD from three independent experiments; **P* < 0.05 (A, B).


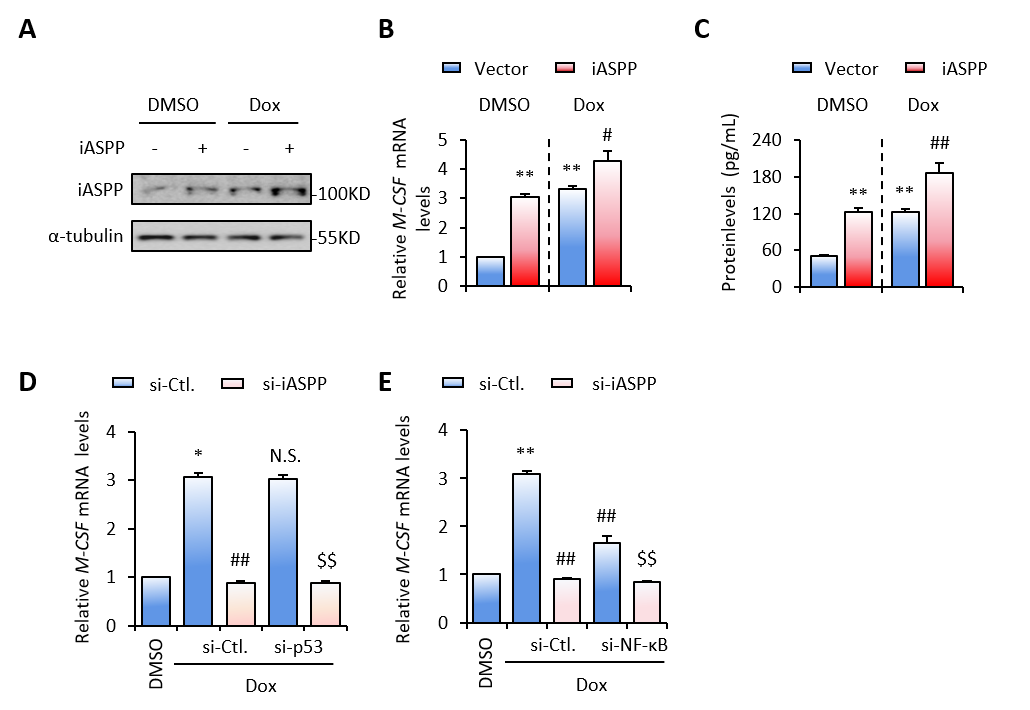


**Figure S2 iASPP-regulated M-CSF expression is mainly dependent on Nrf2**

1. iASPP overexpression (OE) efficiency was confirmed by western blot. (B-C) mRNA and protein levels of M-CSF were determined by qRT-PCR (B) and ELISA (C), respectively, after triggering senescence in iASPP OE HCT116 cells (B, C). (D) mRNA expressions of *M-CSF* were detected by RT-PCR in HCT116 cells after iASPP KD and/or p53 KD (D) or NF-κB KD (E). Values are mean ± SD from three independent experiments; **P* < 0.05, ***P* < 0.01, means compared with DMSO (B, C, D, E); #*P* < 0.05, ##*P* < 0.01, means compared with Dox-treated control (B, C, D, E); $$*P*< 0.01, means compared with Dox and KD p53/ NF-κB -treated control (C, E); N.S., not significant(D).


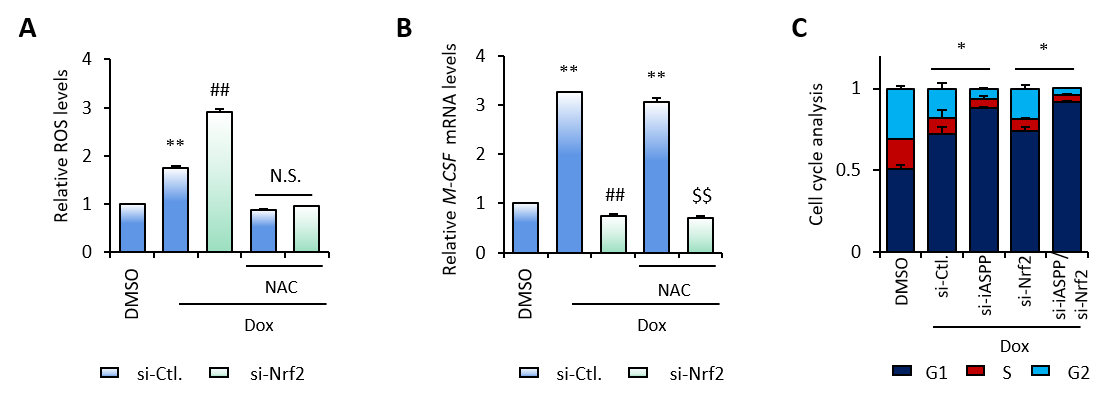


**Figure S3 Nrf2 regulates M-CSF expression independently of ROS**

(A, B) Relative ROS levels (A) and mRNA expression levels of *M-CSF* (B) were detected by flow cytometry and qRT-PCR, respectively, after triggering senescence in Nrf2 KD HCT116 cells in the presence of absence of NAC. (C) The cell cycle distribution was detected by flow cytometry in HCT116 cells after iASPP and/or Nrf2 KD under the control and senescent conditions. Values are mean ± SD from three independent experiments; **P*< 0.05, ***P*< 0.01, means compared with DMSO (A, B, C); ##p < 0.01, means compared with Dox-treated control (A, B); $$*P* < 0.01, means compared with Dox, NAC and KD Nrf2-treated control (B); N.S., not significant(A).


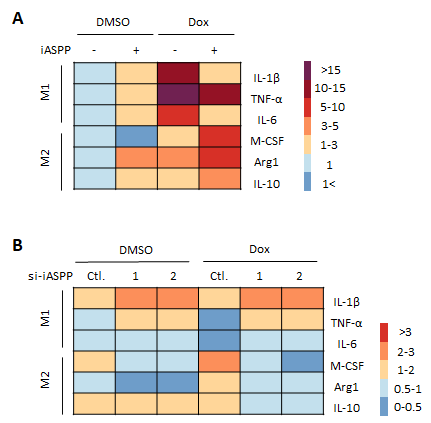


**Figure S4 iASPP promotes M2 polarization**

mRNA levels of M1 markers and M2 markers were detected by qRT-PCR in M0 macrophage cells co-cultured with the conditioned medium of HCT116 cells after OE iASPP (A) and KD iASPP (B) before and after Dox treatment.


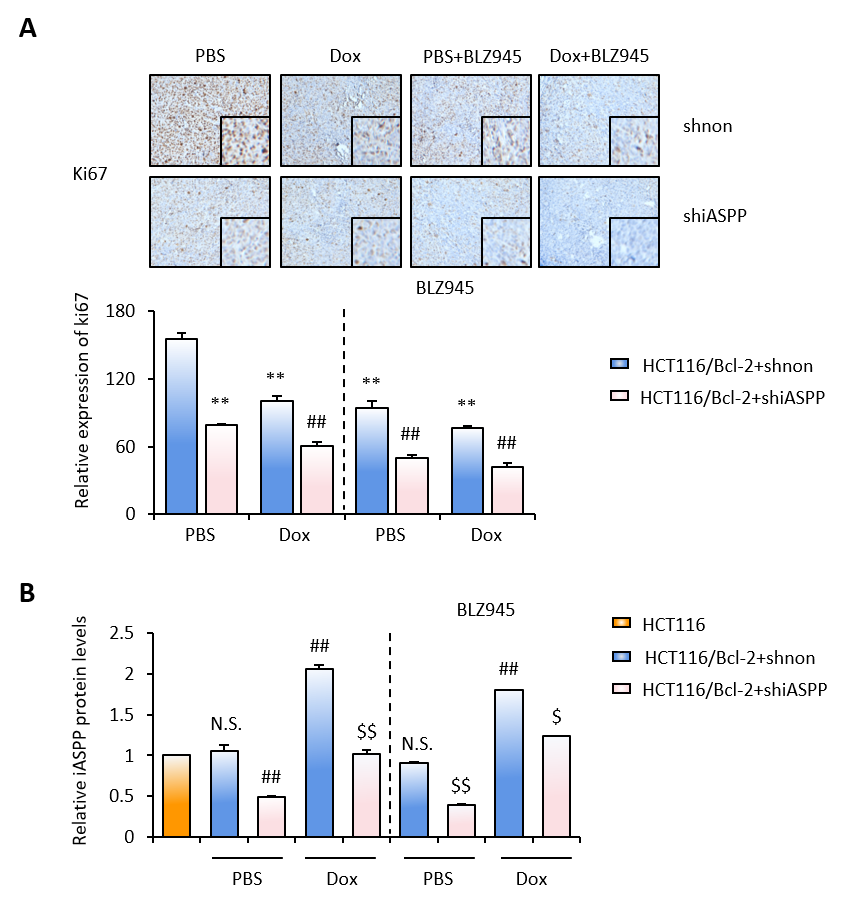


**Figure S5 iASPP KD enhances the sensitivity to Dox**

(A) Representative images of immunohistochemistry staining of Ki67 in the indicated xenografts (up). Expression of ki67 was quantified and shown in bar graph. The iASPP quantitative analysis of the *in vivo*-tumors were shown in (B). Values are mean ± SD; ***P* < 0.01, means compared with PBS control(A); ##*P* < 0.01, means compared with Dox and/or BLZ945-treated control (A), compared with PBS control(B); $*P* < 0.05, $$*P* < 0.01, means compared with Dox or PBS and/or BLZ945-treated control(B); N.S., not significant (B).
